# COVID-19 Vaccination as a Potential Trigger for New-Onset Systemic Lupus Erythematosus

**DOI:** 10.7759/cureus.21917

**Published:** 2022-02-04

**Authors:** Ikwinder Kaur, Saira Zafar, Eugenio Capitle, Reena Khianey

**Affiliations:** 1 Internal Medicine, Monmouth Medical Center, Long Branch, USA; 2 Allergy and Immunology, Rutgers University New Jersey Medical School, Newark, USA; 3 Rheumatology, Rutgers University New Jersey Medical School, Newark, USA

**Keywords:** systemic lupus erythematosus, immunization, autoimmunity, covid-19 vaccination, covid-19

## Abstract

Immune hyperactivation has been linked to various vaccines. We present a potential association of new-onset systemic lupus erythematosus (SLE) post-COVID-19 immunization. The patient is a 54-year-old male admitted for evaluation of flu-like symptoms two weeks after receiving the second dose of the COVID-19 vaccine. Physical examination revealed high-grade fever, diffuse bilateral non-tender cervical lymphadenopathy, and erythematous maculopapular palpable purpuric lesions on bilateral feet. Laboratory evaluation showed a significant hypocomplementemia (C3 < 11 mg/dL, C4 < 3 mg/dL, and CH50 < 10 U/mL), high titer antinuclear antibody, anti-dsDNA antibodies, anti-Sjogren’s syndrome-related antigen A antibodies, anti-Sjogren’s syndrome-related antigen B antibodies, anti-Smith antibodies, anti-ribonucleoprotein antibodies, anti-histone antibodies with a negative malignancy, and infection workup. The patient was treated with a high dose of steroids with a positive response. This case highlights the possibility of SLE, a rare adverse event following COVID-19 vaccination.

## Introduction

Vaccinations are considered a safe and effective way to prevent severe viral illnesses and reduce the spread of infection in patients with autoimmune diseases. Regarding COVID-19, three vaccines are authorized for emergency use in the United States: mRNA vaccines (Pfizer and Moderna) and viral vector vaccines (Johnson & Johnson (J&J)). On August 23, 2021, the US Food and Drug Administration (FDA) approved the first COVID-19 vaccine (Pfizer-BioNTech) for individuals ≥ 16 years of age. The first vaccination round was administered in December 2020. As of December 22, 2021, a total of 62% of the population has been fully vaccinated [1]. The results from safety surveillance performed by the CDC are reassuring. First-month vaccine monitoring has revealed that 90.9% of people had nonserious adverse events including local or systemic reactions. Until January 2022, the reported serious adverse effects include anaphylaxis (approximately five cases per million doses administered), myocarditis, pericarditis, thrombosis with thrombocytopenia syndrome (TTS), Guillain-Barré syndrome (GBS), and rarely death. TTS was confirmed in 57 cases following Johnson & Johnson (J&J) and three cases following the Moderna vaccine; myocarditis was confirmed in 1,233 cases, mostly following mRNA vaccines. There are 302 preliminary reports of GBS following the J&J vaccine after more than 18 million vaccine doses were administered [2,3]. There is limited data on the emergence of systemic lupus erythematosus (SLE) with the COVID-19 vaccine [4]. In this case report, we have presented a patient with a history of well-controlled Sjogren’s syndrome diagnosed with systemic lupus erythematosus after receiving the second dose of the COVID-19 vaccine.

## Case presentation

**Figure 1:**
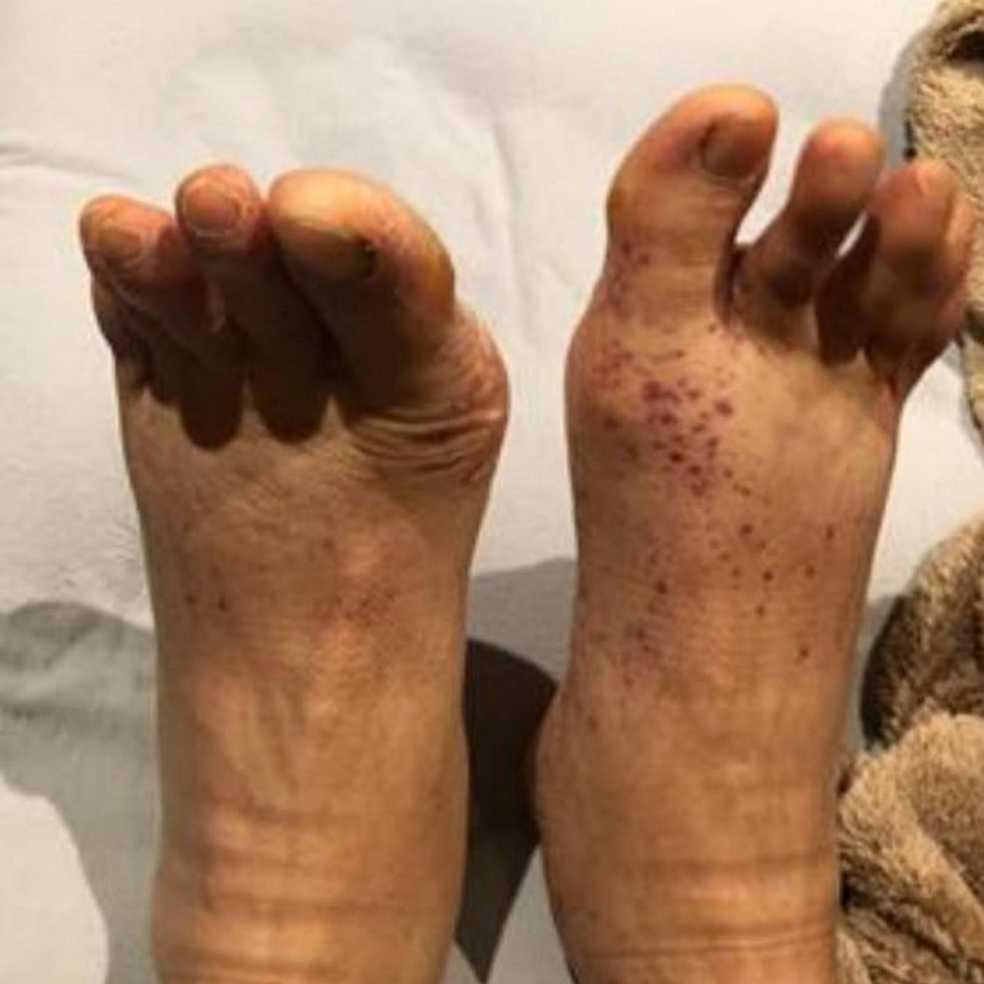
Erythematous maculopapular palpable purpuric lesions on bilateral feet

**Figure 2:**
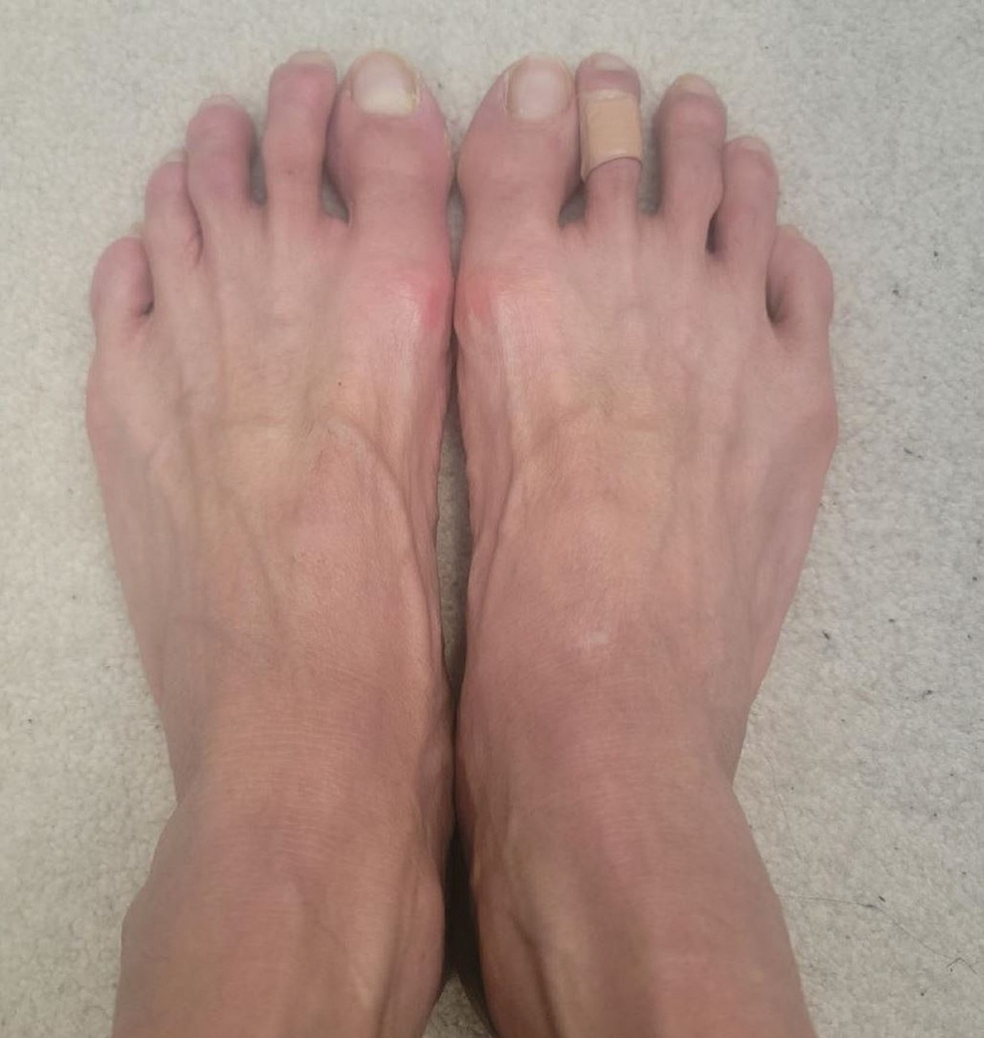
Rash resolution posttreatment

A 54-year-old Asian male was admitted for evaluation of flu-like symptoms two weeks after receiving the second dose of the Pfizer COVID-19 vaccine. The patient complained of fever, fatigue, generalized malaise, loss of appetite, unintentional weight loss, chest heaviness, shortness of breath, worsening of dry mouth and eyes, and burning and pain on bilateral feet. He denied joint pain, swelling or redness, focal weakness, or sensory loss. His only past medical history was stable Sjogren’s syndrome for around 30 years, manifested mainly by keratoconjunctivitis sicca. He was otherwise healthy. The patient denied a history of allergies. His medication list included artificial tears. He was a nonsmoker and occasional alcoholic. His maximum temperature during hospitalization was 101.3°F, and other vital signs remained stable. Physical examination revealed dry mouth, diffuse bilateral non-tender cervical lymphadenopathy, and tachycardia. Skin examination revealed non-pruritic erythematous maculopapular palpable purpuric lesions on the dorsal and plantar surface of the bilateral feet (Figure 1). Other examinations were unremarkable. On laboratory evaluation, the patient was found to have pancytopenia, hyponatremia, hypochloremia, elevated liver function tests, and significant hypocomplementemia (C3 < 11 mg/dL, C4 < 3 mg/dL, and CH50 < 10 U/mL) (Table 1). Malignancy workup including bone marrow biopsy and infection workup including COVID-19 RT-PCR was negative (Table 1). The urinalysis was positive for proteinuria (1.8 g/24 hours), hematuria, and pyuria. CT scan of the chest and abdomen was remarkable for diffuse lymphadenopathy. Chest X-ray and EKG were unremarkable. Additional serologies were positive for high titer antinuclear antibody (1:1,280), anti-dsDNA antibodies (>300 IU/mL), anti-Sjogren’s syndrome-related antigen A antibodies (>8 U/mL), anti-Sjogren’s syndrome-related antigen B antibodies (>8 U/mL), anti-Smith antibodies (>8.0 U/mL), anti-ribonucleoprotein antibodies (7.8 U), anti-histone antibodies (5.2 U), and anti-chromatin antibodies (>8 U/mL). Perinuclear and cytoplasmic antineutrophil cytoplasmic antibodies (P-ANCA and C-ANCA) were negative. According to the American College of Rheumatology (ACR) and Systemic Lupus International Collaborating Clinics (SLICC) criteria, the patient was classified as having SLE. Considering an evolution to SLE, the patient was started on prednisone 60 mg daily and mycophenolate mofetil 1,000 mg daily. He refused a renal biopsy. He improved significantly with the resolution of fever, fatigue, malaise, skin rash (Figure 2), pancytopenia, and transaminitis after initiation of treatment. Proteinuria went down to 500 mg/24 hours. After around a week, he developed confusion and recent memory amnesia. The neurology examination was non-focal. Lumbar puncture to rule out CNS infection, head CT, brain MRI, and EEG were unremarkable. He was started on Solu-Medrol 1,000 mg daily for three days, and mycophenolate mofetil was optimized to 3 g daily for the treatment of possible neuropsychiatric lupus. After two weeks of treatment, the patient stated a significant improvement of neuropsychiatric manifestations.

**Table 1:** Laboratory results L: low value; H: high value; WBC: white blood count; aPTT: activated partial thromboplastin clotting time; INR: international normalized ratio; AST: aspartate aminotransferase; ALT: alanine aminotransferase; CRP: C-reactive protein; ESR: erythrocyte sedimentation rate; ANA: antinuclear antibody; SSA: Sjogren’s syndrome-related antigen A; SSB: Sjogren’s syndrome-related antigen B; CCP: cyclic citrullinated peptides; TB: tuberculosis; HIV: human immunodeficiency virus; HTLV: human T-lymphotropic virus; EBV: Epstein–Barr virus

Laboratory test	Value	Normal values
Complete blood count		
WBC count	1,700/mm^3^ (L)	4,500–11,000/mm^3^
Hemoglobin	9.2 g/dL (L)	13.5–17.5 g/dL
Platelet count	77,000/μL (L)	135,000– 317,000/μL
Absolute neutrophil count	1,400/μL (L)	>1,500/μL
Absolute monocyte count	100/μL (L)	200–800/μL
Absolute lymphocyte count	200/μL (L)	1,000–4,800/μL
Absolute eosinophil and basophil count	0	<500/μL
Peripheral smear	There is pancytopenia. Red blood cells do not show any significant morphological abnormality. Platelet count is reduced in number. White blood cells do not show any blasts. Granulocytes show some blasts with the left shift.	-
CD4/CD8 ratio	0.9	>1
Coagulation profile		
aPTT	44 seconds (H)	25–35 seconds
INR	1.06	<1.1
Prothrombin time	13.5 seconds	11–13.5 seconds
Comprehensive metabolic panel		
Sodium	122 mmol/L ( L)	136–145 mEq/L
Potassium	4.1 mmol/L	3.5–5.0 mEq/L
Chloride	94 mmol/L (L)	95–105 mEq/L
Fasting glucose	89 mg/dL	70–110 mg/dL
Albumin	2.3 g/dL ( L)	3.5–5.5 g/dL
Bilirubin, total	0.3 mg/dL	0.1–1.0 mg/dL
Calcium	7.1 mg/dL ( L)	8.4–10.2 mg/dL
Creatinine	0.8 mg/dL	0.6–1.2 mg/dL
AST	59 U/L (H)	8–40 U/L
ALT	28 U/L	8–40 U/L
Other laboratory values		
Ferritin	3,223 μg/L (H)	15–200 ng/mL
Triglyceride	152 mg/dL (H)	35–160 mg/dL
24-hour urine protein	1,830 mg (H)	<150 mg
Ceruloplasmin	20 mg/dL	14–40 mg/dL
Cortisol, baseline	14.4 μg/dL	5–23 μg/dL
TSH	1.5 μU/mL	0.5–5 μU/mL
Rheumatologic workup		
CRP	0.8 mg/dL	0.0–0.8 mg/dL
ESR	59 mmHg (H)	0–15 mm/hour
ANA screen	Positive	Negative
ANA titer	1:1,280 (H)	<1:160
ANA pattern	Nuclear, speckled	-
C4 complement level	<3 mg/dL ( L)	10–40 mg/dL
C3 complement level	<11 mg/dL (L)	55–120 mg/dL
Total complement (CH50)	<10 U/mL ( L)	37–55 U/mL
Anticardiolipin antibody	Negative	Negative
Antinuclear ribonucleoprotein antibodies	7.8 U (positive)	Negative
Anti-Smith antibodies	>8.0 U/mL (positive)	Negative
SSA	>8 U/mL (positive)	Negative
SSB	>8 U/mL (positive)	Negative
Anti-DNA antibodies	>300 IU/mL (positive)	Negative
Anti-chromatin antibodies	>8 U/mL (positive)	Negative
Anti-histone antibodies	5.2 U (positive)	Negative
Beta-2-glycoprotein 1	Negative	Negative
Lupus anticoagulant	Negative	Negative
Anti-centromere antibodies	Negative	Negative
Anti-Jo-1 antibodies	Negative	Negative
Rheumatoid factor	Negative	<40 U/mL
Anti-CCP	Low titer	Negative
Cryoglobulins	Negative	Negative
Malignancy workup		
Occult blood stool	Negative	Negative
Urine protein electrophoresis	No monoclonal protein identified	-
Bone marrow biopsy	Peripheral cytopenia of undetermined significance. Bone marrow showing maturing trilineage hematopoiesis with normal cellularity for age. No acute leukemia, lymphoma, or high-grade clonal stem cell disorder was identified.	-
Flow cytometry results	Viability is 86%. Lymphocytes comprise 21% of the sample, granulocytes 59%, and monocytes 1%. No increased blasts are identified. No monoclonal B lymphoid population is identified. No dropped pan T-cell antigens are identified. No significantly abnormal myeloid antigen expression is identified.	-
Final diagnosis	Peripheral blood smear showing pancytopenia. Flow cytometry does not identify any leukemia, lymphoma, or high-grade clonal stem cell disorder.	-
Infection workup		
SARS-CoV-2 antibody total	Positive	Negative
Respiratory pathogen panel by RT-PCR with COVID-19 (COVID-19, influenza, parainfluenza, RSV, *Bordetella pertussis*, *Bordetella parapertussis*, *Chlamydia pneumoniae*, *Mycoplasma pneumoniae*, adenovirus, metapneumovirus, rhinovirus, enterovirus)	Not detected	Negative
Legionella antigen, urine	Negative	Negative
Ehrlichia antibody	Not detected	Negative
Lyme antibody screen	Negative	Negative
Chlamydia antibody panel	Not detected	Negative
Hepatitis acute panel	Not detected	Negative
QuantiFERON-TB Gold	Indeterminate	Negative
Rapid HIV	Non- reactive	Negative
HTLV antibody screen	Not detected	Negative
Cytomegalovirus antibody ( IgM)	Not detected	Negative
EBV antibody titers	Suggestive of a past EBV infection	Negative
Parvovirus B19 antibody titer	Negative	Negative
Malaria/Babesia smear	Negative	Negative
Blood culture	Negative	Negative

## Discussion

COVID-19 infection is associated with a flare-up of preexisting autoimmune rheumatic diseases or new-onset autoimmunity [5]. However, data regarding immune hyperactivation causing a disease flare or new onset of an autoimmune disorder post-COVID-19 vaccination is limited. Patients with autoimmune disorders were excluded from the vaccine trials, and guidelines regarding COVID-19 vaccination are based on expert opinion. There is a scarcity of long-term safety data in this patient population. A recent study in patients with SLE has assessed a mild to moderate disease flare in 11.4% of patients and severe flare in 1.3% of patients post-COVID-19 vaccination [6]. Hepatitis B, HPV, and influenza vaccines have increased the risk of autoimmunity as mentioned in a meta-analysis performed by Wang et al. [7]. The etiology is unclear; the proposed mechanism can be that inactive viral component or attenuated microorganism induces molecular mimicry or bystander activation in genetically predisposed individuals. Also, adjuvants added to enhance immunity in various vaccines can result in autoimmune events. Given the negative infectious and malignancy workup, a temporal relationship with COVID-19 vaccination, the biological plausibility of the vaccines causing autoimmunity, and clinical characteristics, the COVID-19 vaccine was considered as a possible trigger for the evolution of SLE in our patient. Skin lesions were thought to be secondary to possible lupus vasculitis. The cutaneous biopsy was deferred because of self-resolution with the treatment of SLE. This report illustrates that although the COVID-19 vaccine is considered safe to prevent the spread of disease among patients with preexisting autoimmune diseases, rare disease flares or evolution to a new autoimmune disease can be triggered. Nationwide studies demonstrating long-term COVID-19 vaccine safety in patients with preexisting well-controlled autoimmune diseases are needed to support the finding.

## Conclusions

This case highlights the possibility of systemic lupus erythematosus, a rare adverse event following COVID-19 vaccination. Early recognition and treatment can prevent potentially serious complications related to SLE post-vaccination. Although the COVID-19 vaccine is highly recommended by experts in patients with autoimmune diseases, results from high-quality studies are warranted to demonstrate long-term safety in this patient population.
